# Viscous flow between two sinusoidally deforming curved concentric tubes: advances in endoscopy

**DOI:** 10.1038/s41598-021-94682-8

**Published:** 2021-07-23

**Authors:** L. B. McCash, Salman Akhtar, Sohail Nadeem, Salman Saleem, Alibek Issakhov

**Affiliations:** 1 School of Mathematics and Actuarial Science, Univerisity of Leicester, Leicester, LE1 7RH UK; 2 Department of Mathematics, Quaid-i-Azam University 45320, Islamabad, 15320 Pakistan; 3 Department of Mathematics, College of Science King Khalid University, P. O. Box 9004, Abha, 61413 Saudi Arabia; 4 Faculty of Mechanics and Mathematics, Al-Farabi Kazakh National University, av. al-Farabi 71, Almaty, Kazakhstan

**Keywords:** Psychology, Mathematics and computing

## Abstract

Viscous flow between two sinusoidally deforming curved concentric tubes is mathematically investigated for the first time. Exact solutions are computed to analyse the flow between these two tubes and graphical outcomes are included for a thorough analysis of the solutions. The present article has prime applications in endoscopy as a novel peristaltic endoscope is introduced first time for a curved sinusoidal tube. This curved nature of outer sinusoidal tube with a flexible peristaltic endoscope placed inside it covers the topic of practical applications like endoscopy of human organs having curved shapes and the maintenance of complex machineries that involve complex curve structures. The usage of a flexible peristaltic endoscope inside a curved sinusoidal tube makes the process of catheterization more comfortable.

## Introduction

Endoscopy is the mechanism that is utilized to examine the internal cavities of human body and it is also useful for repairing and maintenance of complex machineries. In order to meet the complex scenarios like catheterization in the human body and the restoration of machines having complex internal structures, it is very important to have a flexible endoscope rather than a rigid one. Such a flexible endoscope can easily move through curve tubes and corners while the rigid one can damage the internal cavities of body and also causes discomfort to the patient in some cases. Thus a flexible endoscope has wide range of applications both in the medicine and industry. Further, it is more useful to have a flexible endoscope that has the same shape as the shape of the cavity in which it is being inserted, since it makes the process of catheterization even more comfortable. For such practical purposes, a novel endoscope that is called a peristaltic endoscope (an endoscope having deformable sinusoidal walls) is developed and its locomotion is experimentally tested^1^. Such a peristaltic endoscope consists of McKibben-like actuators around the main hollow tube and each of the actuator has its own source of compressed air for expansion and a spring for contraction as well. This novel peristaltic endoscope is useful for the endoscopy of tubes that also have deformable sinusoidal walls. Rachid et al.^2–4^ had first time used this concept of peristaltic flow in a tube also having a peristaltic endoscope in it.

Peristaltic flow activity occurs within a tube having deformable sinusoidal walls which propagate the flow due to their rhythmic sinusoidal contraction and relaxation. This peristalsis mechanism is an inherited feature of the smooth tubular shape muscles present in human body. Thus it has a key role in some physiological activities that include vasomotion, functioning of ureter, gastrointestinal tract and bile duct etc^5^. The mathematical analysis of fluid transportation inside a tube due to deforming sinusoidal walls was first time presented by Barton and Raynor^6^. Pozrikidis^7^ had utilized the creeping flow approximations for peristaltic flow in two dimensional geometries. Since our present study deals with the peristaltic flow inside a curved tube, therefore some useful and recent researches that interpret the mathematical analysis of peristaltic flow inside curved tubes are referred as^8–15^. Further, the analysis of endoscopy for peristaltic flow inside a tube is also carried out by many researchers^16–19^ and some of the recent mathematical interpretations are also given as^20–23^.

We here disclose a mathematical investigation that first time interprets the peristaltic flow inside a curved tube having a flexible peristaltic endoscope in it. This curved tube has deforming sinusoidal walls and also the peristaltic endoscope placed in it has deformable sinusoidal walls. The selection of a curved sinusoidal tube for this mathematical analysis covers the practical applications like endoscopy of flexible curved tubes present in human body and maintenance of complex machineries that involve curved structures etc. Moreover, a flexible peristaltic endoscope having sinusoidally deforming walls is placed in this sinusoidal curved tube to achieve the recent advance targets in the field of endoscopy.

## Mathematical model

**Figure 1:**
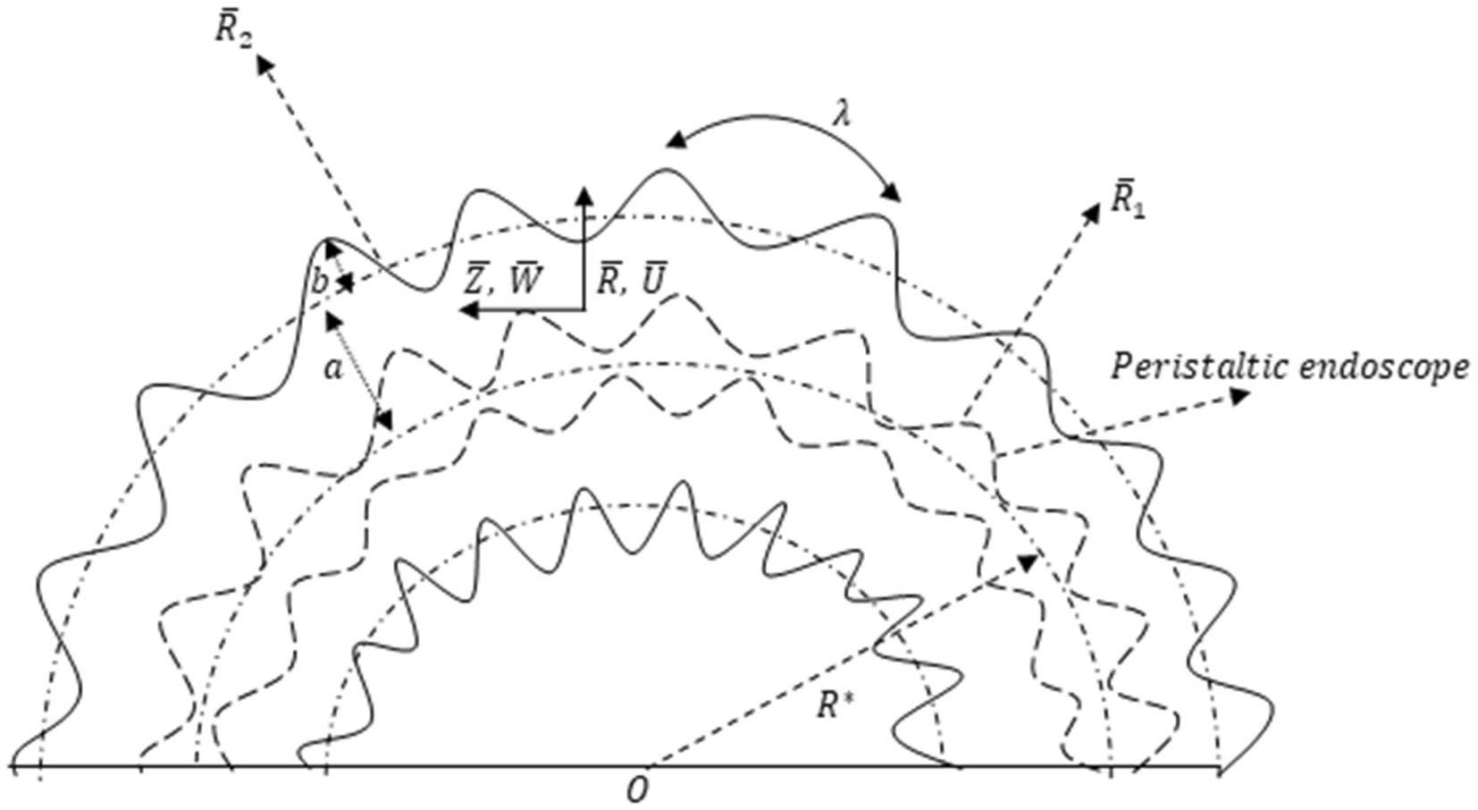
Geometrical model of the problem.

An incompressible, viscous flow between two sinusoidally deforming curved concentric tubes is mathematically interpreted. Both of these tubes have sinusoidally deforming walls, as shown in Fig. 1.

The mathematical representation of both these (i.e. outer and inner) sinusoidally deforming walls is narrated as

1 $$\begin{aligned} \overline{R}_{2} & = a + bSin\frac{2\pi }{\lambda }\left( {\overline{Z} - c\overline{t}} \right), \\ \overline{R}_{1} & = n\overline{R}_{2} = na + nbSin\frac{2\pi }{\lambda }\left( {\overline{Z} - c\overline{t}} \right), \\ \end{aligned}$$

where $0 < n < 1$. Since $n = 0$ means that there is no catheter present in the tube and for $n = 1$ means that the catheter has a radius equal to that of outer tube’s radius. Both of these cases are not of our interest that is why we have selected $0 < n < 1$.

A dimensional mathematical representation of the governing differential equations are achieved by utilizing a curvilinear coordinate system and narrated as^15^

2 $$\frac{\partial }{{\partial \overline{R}}}\left( {\left( {\overline{R} + R^{*} } \right)\overline{U}} \right) + R^{*} \frac{{\partial \overline{W}}}{{\partial \overline{Z}}} = 0,$$

3 $$\begin{aligned} \rho \left( {\frac{{\partial \overline{U}}}{{\partial \overline{t}}} + \overline{U}\frac{{\partial \overline{U}}}{{\partial \overline{R}}} + \frac{{R^{*} \overline{W}}}{{\overline{R} + R^{*} }}\frac{{\partial \overline{U}}}{{\partial \overline{Z}}} - \frac{{\overline{W}^{2} }}{{\overline{R} + R^{*} }}} \right) & = - \frac{{\partial \overline{P}}}{{\partial \overline{R}}} + \mu \left[ {\frac{1}{{\left( {\overline{R} + R^{*} } \right)}}\frac{\partial }{{\partial \overline{R}}}\left\{ {\left( {R^{*} + \overline{R}} \right)\frac{{\partial \overline{U}}}{{\partial \overline{R}}}} \right\}} \right. \\ & \quad \left. { + \left( {\frac{{R^{*} }}{{R^{*} + \overline{R}}}} \right)^{2} \frac{{\partial^{2} \overline{U}}}{{\partial \overline{Z}^{2} }} - \frac{{\overline{U}}}{{\left( {R^{*} + \overline{R}} \right)^{2} }} - \frac{{2R^{*} }}{{\left( {R^{*} + \overline{R}} \right)^{2} }}\frac{{\partial \overline{W}}}{{\partial \overline{Z}}}} \right] \\ \end{aligned}$$

4 $$\begin{aligned} \rho \left( {\frac{{\partial \overline{W}}}{{\partial \overline{t}}} + \overline{U}\frac{{\partial \overline{W}}}{{\partial \overline{R}}} + \frac{{R^{*} \overline{W}}}{{\overline{R} + R^{*} }}\frac{{\partial \overline{W}}}{{\partial \overline{Z}}} + \frac{{\overline{W}\overline{U}}}{{\overline{R} + R^{*} }}} \right) & = - \left( {\frac{{R^{*} }}{{\overline{R} + R^{*} }}} \right)\frac{{\partial \overline{P}}}{{\partial \overline{Z}}} + \mu \left[ {\frac{1}{{\left( {\overline{R} + R^{*} } \right)}}\frac{\partial }{{\partial \overline{R}}}\left\{ {\left( {R^{*} + \overline{R}} \right)\frac{{\partial \overline{W}}}{{\partial \overline{R}}}} \right\}} \right. \\ & \quad \left. { + \left( {\frac{{R^{*} }}{{R^{*} + \overline{R}}}} \right)^{2} \frac{{\partial^{2} \overline{W}}}{{\partial \overline{Z}^{2} }} - \frac{{\overline{W}}}{{\left( {R^{*} + \overline{R}} \right)^{2} }} + \frac{{2R^{*} }}{{\left( {R^{*} + \overline{R}} \right)^{2} }}\frac{{\partial \overline{U}}}{{\partial \overline{Z}}}} \right] \\ \end{aligned}$$

The two distinct reference frames (fixed and moving) are linked with the help of following transformation equations narrated as

5 $$\overline{z} = \overline{Z} - c\overline{t}, \overline{r} = \overline{R}, \overline{p} = \overline{P} ,\overline{w} = \overline{W} - c, \overline{u} = \overline{U},$$

The useful dimensionless terms that appeared during the mathematical computations are narrated as

6 $$r = \frac{{\overline{r}}}{a}, z = \frac{{\overline{z}}}{\lambda }, w = \frac{{\overline{w}}}{c}, u = \frac{{\lambda \overline{u}}}{ac}, p = \frac{{a^{2} \overline{p}}}{c\lambda \mu }, \beta = \frac{a}{\lambda },\user2{ }s = \frac{{R^{*} }}{a}, \phi = \frac{b}{a}, r_{1} = \frac{{\overline{R}_{1} }}{a}, r_{2} = \frac{{\overline{R}_{2} }}{a},$$

The dimensionless representation of the present mathematical problem is achieved by utilizing the transformation Eqs. (5) and then the non-dimensional terms narrated in Eq. (6), into Eqs. (2–4) and we get after using $\lambda \to \infty$ or $R_{e} \to 0$*.*

7 $$\frac{\partial p}{{\partial r}} = 0,$$

8 $$\left( {\frac{s}{r + s}} \right)\frac{\partial p}{{\partial z}} = \frac{1}{{\left( {r + s} \right)}}\frac{\partial }{\partial r}\left\{ {\left( {s + r} \right)\frac{\partial w}{{\partial r}}} \right\} - \frac{{\left( {w + 1} \right)}}{{\left( {s + r} \right)^{2} }},$$

The corresponding boundary conditions for this problem are

9 $$\begin{aligned} & w = - 1\quad {\text{at}}\quad r = r_{1} = n + n\phi Sin\left( {2\pi z} \right), \\ & w = - 1\quad {\text{at}}\quad r = r_{2} = 1 + \phi Sin\left( {2\pi z} \right), \\ \end{aligned}$$

## Exact solution

We have computed exact mathematical results for this present problem by utilizing Mathematica software. The velocity solution provided below is attained by solving Eq. (8) along with conditions given in Eq. (9).

10 $$w = - 1 + \frac{{\left[ {\frac{\partial p}{{\partial z}}s\left\{ {\left( {r_{1} - r_{2} } \right)\left( {r + s} \right)^{2} \left( {r_{1} + r_{2} + 2s} \right)Log\left( {r + s} \right) - \left( {r - r_{2} } \right)\left( {r_{1} + s} \right)^{2} \left( {r + r_{2} + 2s} \right)Log\left( {r_{1} + s} \right) + \left( {r - r_{1} } \right)\left( {r_{2} + s} \right)^{2} \left( {r + r_{1} + 2s} \right)Log\left( {r_{2} + s} \right)} \right\}} \right]}}{{\left( {2\left( {r_{1} - r_{2} } \right)\left( {r + s} \right)\left( {r_{1} + r_{2} + 2s} \right)} \right)}}$$

Further, the rate of volumetric flow is narrated by Eq. (11) as follows

11 $$F = \mathop \smallint \limits_{{r_{1} }}^{{r_{2} }} r.wdr,$$

The mathematical result for pressure gradient is attained by using the above Eq. (11) and narrated as

12 $$\frac{dp}{{dz}} = \frac{{\left( {36\left( {r_{1} - r_{2} } \right)\left( {2F - r_{1}^{2} + r_{2}^{2} } \right)\left( {r_{1} + r_{2} + 2s} \right)} \right)}}{{\left[ {s\left\{ {\left( {r_{1} - r_{2} } \right)^{2} \left( {r_{1} + r_{2} + 2s} \right)\left( {4\left( {r_{1}^{2} + r_{1} r_{2} + r_{2}^{2} } \right) + 3\left( {r_{1} + r_{2} } \right)s - 6s^{2} } \right) - 12\left( {r_{1} + s} \right)^{2} \left( {r_{2} + s} \right)^{2} \left( {Log\left( {r_{1} + s} \right) - Log\left( {r_{2} + s} \right)} \right)\left( {2r_{1} - 2r_{2} - 3sLog\left( {r_{1} + s} \right) + 3sLog\left( {r_{2} + s} \right)} \right)} \right\}} \right]}}$$

where $Q = F + 2$ and the pressure rise is computed numerically over single wavelength by utilizing the following equation given as

13 $$\Delta P = \mathop \smallint \limits_{0}^{1} \frac{\partial p}{{\partial z}}dz,$$

## Results and discussion

**Figure 2:**
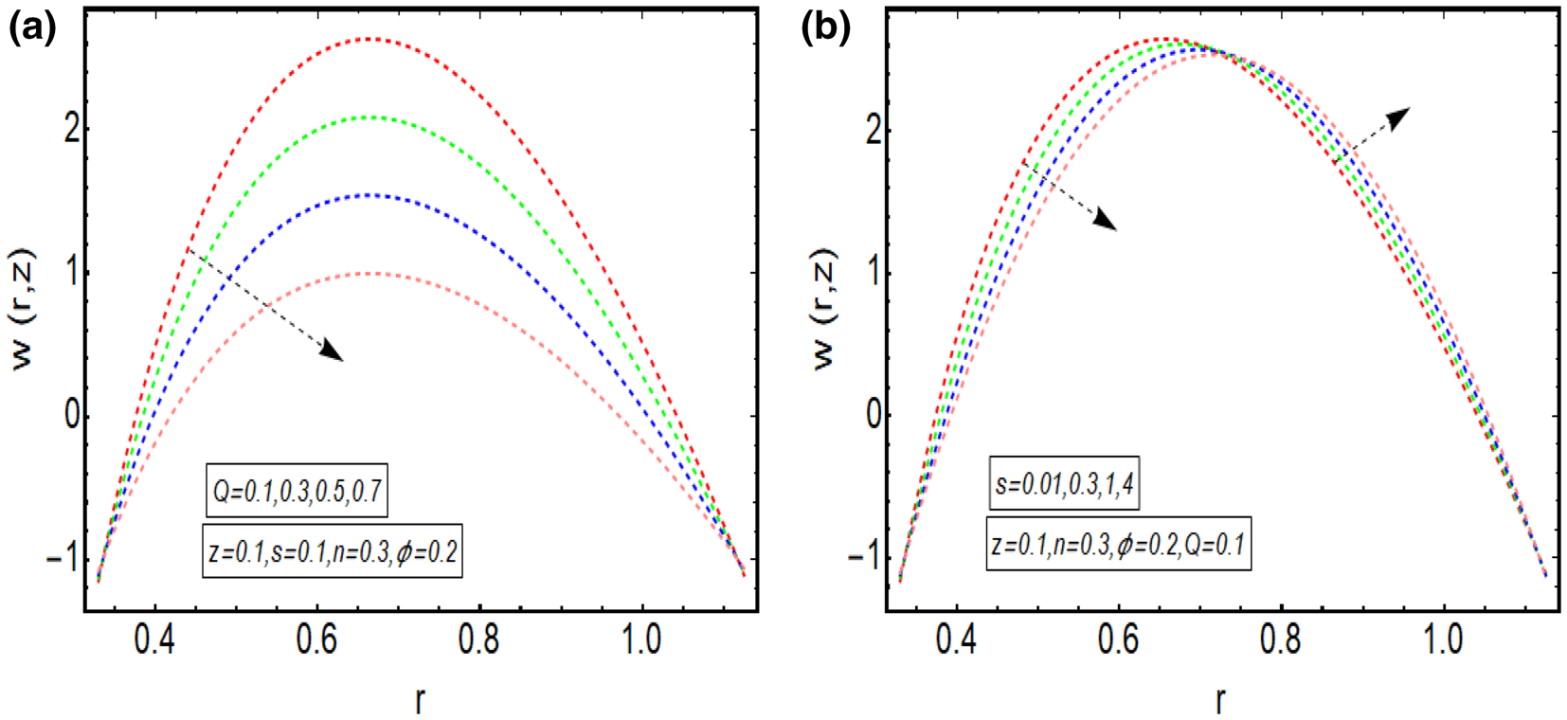
(**a**) Velocity plot for four values of $Q.$ (**b**) Velocity plot for four values of $s$.

**Figure 3:**
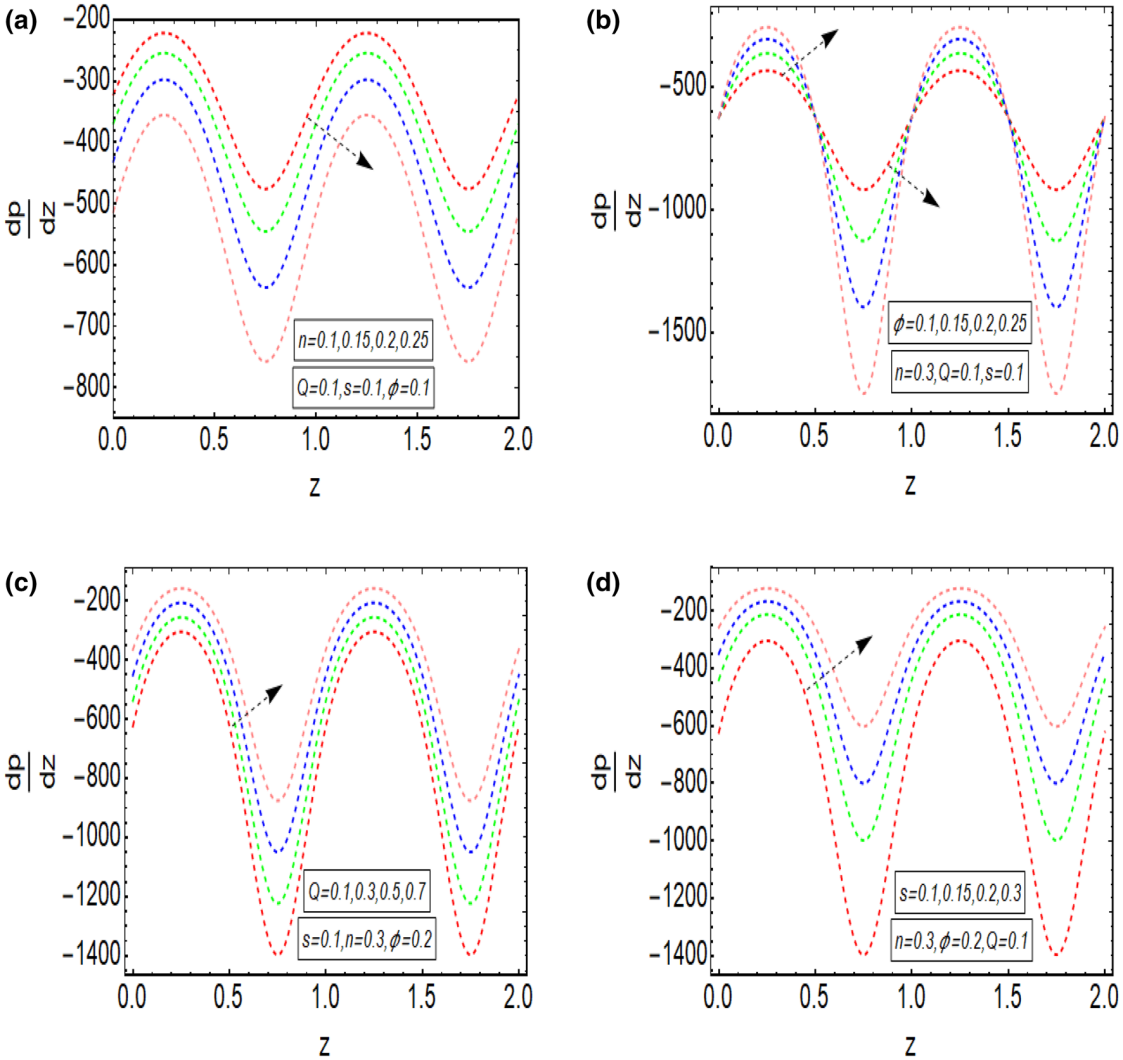
(**a**) $\frac{dp}{{dz}}$ plot for four values of $n$. (**b**) $\frac{dp}{{dz}}$ plot for four values of $\phi$. (**c**) $\frac{dp}{{dz}}$ plot for four values of $Q$. (**d**) $\frac{dp}{{dz}}$ plot for four values of $s$.

**Figure 4:**
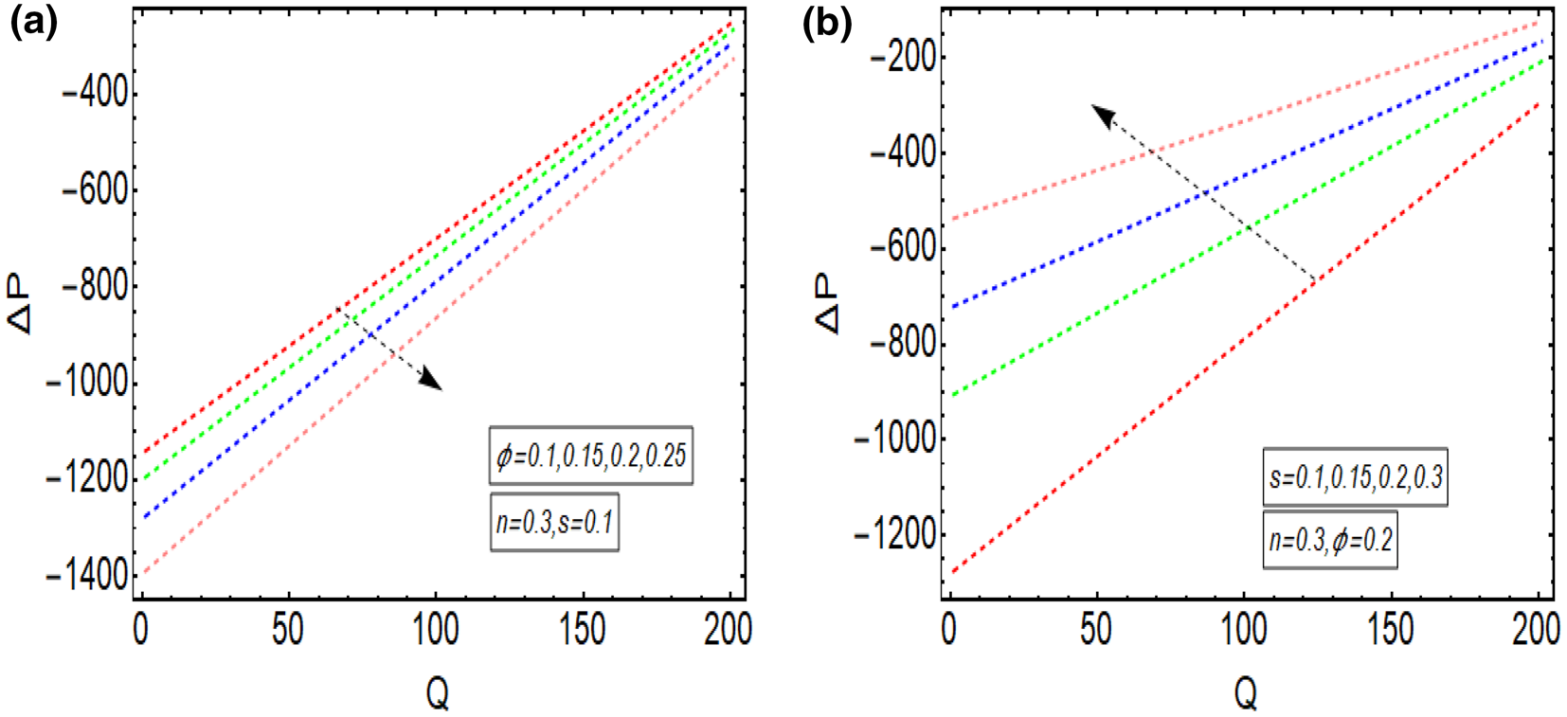
(**a**) $\Delta P$ plot against $Q$ for four values of $\phi$. (**b**) $\Delta P$ plot against $Q$ for four values of $s$.

**Figure 5:**
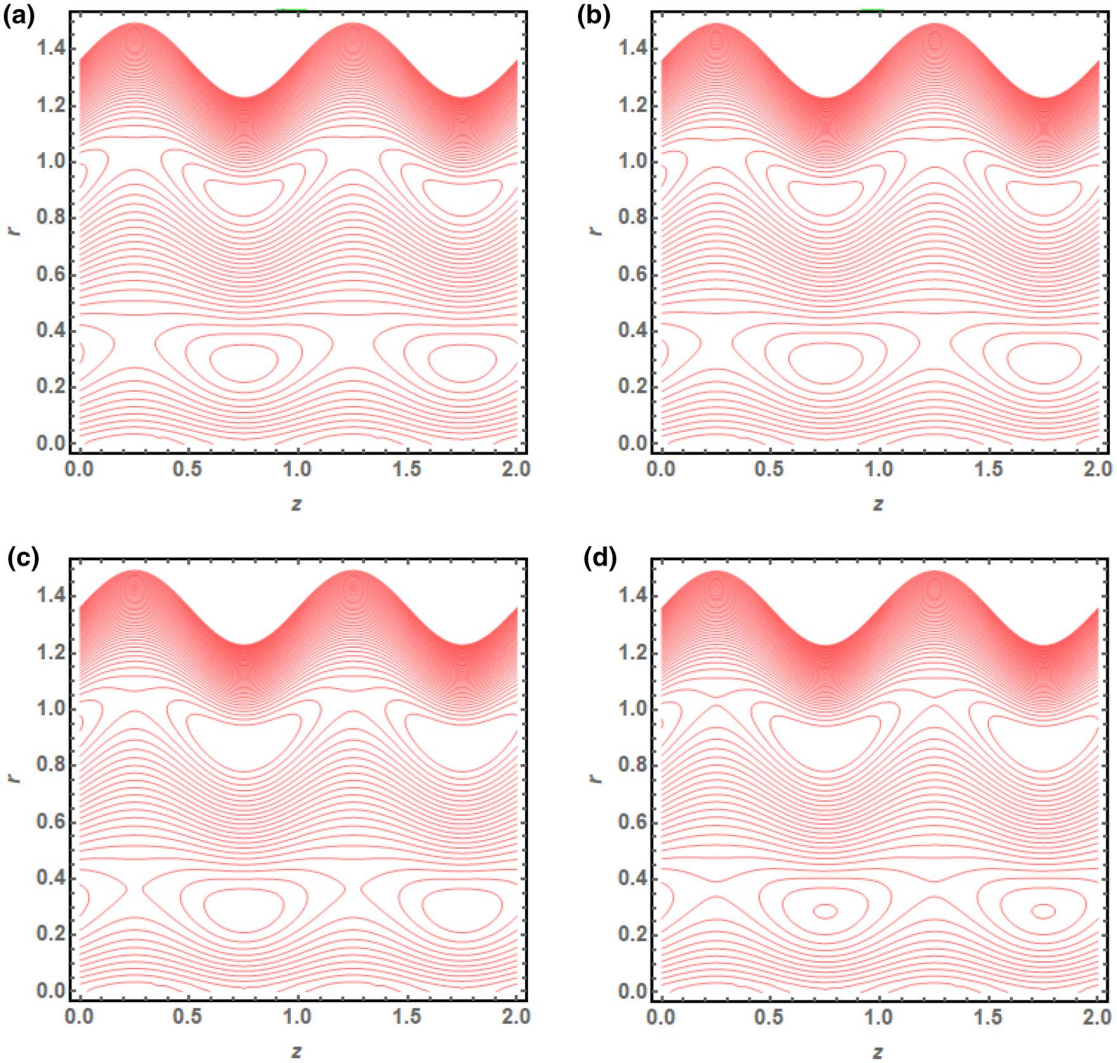
(**a**) Streamline plot for $Q = 0.01$. (**b**) Streamline plot for $Q = 0.03$. (**c**) Streamline plot for $Q = 0.05$. (**d**) Streamline plot for $Q = 0.07$.

**Figure 6:**
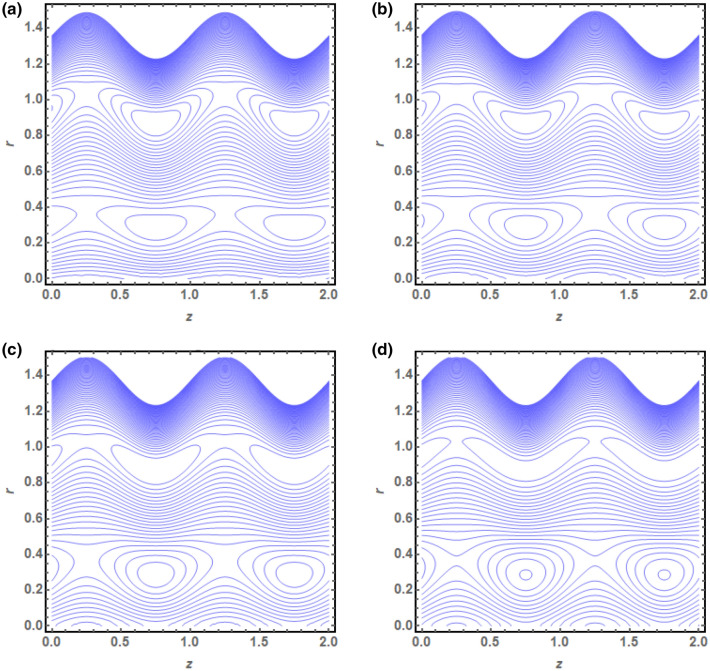
(**a**) Streamline plot for $s = 0.01$. (**b**) Streamline plot for $s = 0.02$. (**c**) Streamline plot for $s = 0.03$. (**d**) Streamline plot for $s = 0.04$.

The above mathematical computations are now illustrated through graphical solutions. These graphical results are computed for the velocity, pressure gradient, pressure rise and streamlines of this flow problem. Figure 2a,b present the graphical results of velocity for the non-dimensional parameters $Q$ and $s$ respectively. A reduction in the velocity is observed with increasing $Q$, revealed in Fig. 2a. Thus velocity is varying inversely with increasing $Q$ in this curved sinusoidal tube having a peristaltic endoscope in it. Figure 2b provides the graphical result of velocity for varying values of $s$. It is noted that velocity is declining with the peristaltic endoscope wall (i.e. inner sinusoidal wall) but it is increasing with the outer sinusoidal wall of this curved tube for increasing curvature parameter $s$. Figure 3a–d provide the graphical results of $\frac{dp}{{dz}}$ against $z - axis$ for various parameters involved in this study. Figure 3a provides the graphical outcome of $\frac{dp}{{dz}}$ for increasing $n$ (i.e. increasing radius of peristaltic endoscope). Here $\frac{dp}{{dz}}$ is declining with an increase in the radius of peristaltic endoscope. Figure 3b shows that $\frac{dp}{{dz}}$ increments with the evolution of peristaltic wave but it declines with shrinkage of the peristaltic wave for increasing $\phi$. Figure 3c conveys an increase in the value of $\frac{dp}{{dz}}$ for increasing $Q$. An increment is noted in the value of $\frac{dp}{{dz}}$ for increasing curvature parameter $s$, as presented in Fig. 3d. Figure 4a,b provide the graphical solutions of $\Delta P$ against $Q$. A reduction in the value of $\Delta P$ is observed with increasing $\phi$, revealed in Fig. 4a. The numerical value of $\Delta P$ is increasing for higher values of $s$, depicted in Fig. 4b. Figure 5a–d presents the graphical solutions of streamlines plot for increasing $Q$. The outer sinusoidal wall of this curved tube is evident at the upper end of graphical plot and the lower end also shows the sinusoidal pattern due to the presence of peristaltic endoscope. A reduction in the trapping phenomenon is noted for increasing $Q$. Figure 6a–d reveal the graphical results of streamline plot for increasing curvature parameter $s$. These outcomes show that the trapping is declining with the outer sinusoidal wall of curved tube but it is increasing with the wall of the peristaltic endoscope.

## Conclusions

The Newtonian flow between two sinusoidally deforming curved concentric tubes is mathematically investigated. The present analysis has recent endoscopic applications. Some major outcomes of this analysis are as follows.

- The application of a flexible peristaltic endoscope is helpful in the process of catheterization in curved tubes as most of the patients face discomfort due to the usage of rigid or semi-rigid endoscopes.
- The same shape and structure of both the channel and endoscope helps to meet a wide range of medical and industrial applications.
- This peristaltic endoscope model for a curved sinusoidal channel is also useful for complex machineries as it helps in their maintenance.
- This mathematical analysis is a benchmark research for further advances in the field of endoscopy using a flexible peristaltic endoscope that has the same shape and structure as that of the channel in which it is placed.
- A symmetric trapping phenomenon is observed through streamlines plot.

## List of symbols

$a$: Mean radius of curved tube
$R^{*}$: Radius of curved geometrical model
$$\mu$$: Dynamic viscosity
$b$: Amplitude of peristaltic wave
$\beta$: Wave number in dimensionless form
$\overline{R}_{1}$: Dimensional radius of inner tube
$r_{1}$: Dimensionless radius of inner tube
$\left( {\overline{U},\overline{W}} \right)$: Dimensional form of velocity field
$\left( {\overline{R},\overline{Z}} \right)$: Curvilinear coordinate system
$$c$$: Wave speed
$$\lambda$$: Wavelength
$$s$$: Curvature parameter
$$\phi$$: Amplitude ratio
$\overline{R}_{2}$: Dimensional radius of outer tube
$r_{2}$: Dimensionless radius of outer tube
$\left( {u,w} \right)$: Dimensionless form of velocity field
